# Prevalence of sexual dysfunction in women with cancer: A systematic review and meta-analysis

**DOI:** 10.18502/ijrm.v20i1.10403

**Published:** 2022-02-18

**Authors:** Seyedeh Esmat Hosseini, Mahnaz Ilkhani, Camelia Rohani, Alireza Nikbakht Nasrabadi, Raza Ghanei Gheshlagh, Ashraf Moini

**Affiliations:** ^1^Department of Medical Surgical Nursing, School of Nursing and Midwifery, Shahid Beheshti University of Medical Sciences, Tehran, Iran.; ^2^Cancer Research Center, Shahid Beheshati University of Medical Sciences, Tehran, Iran.; ^3^Department of Health Care Sciences, Palliative Care Center, Ersta Sköndal Bräcke University College, Campus Ersta, Stockholm, Sweden.; ^4^Department of Community Health Nursing, School of Nursing and Midwifery, Shahid Beheshti University of Medical Sciences, Tehran, Iran.; ^5^School of Nursing and Midwifery, Tehran University of Medical Sciences, Tehran, Iran.; ^6^Spiritual Health Research Center, Research Institute for Health Development, Kurdistan University of Medical Sciences, Sanandaj, Iran.; ^7^Breast Disease Research Center (BDRC), Tehran University of Medical Sciences, Tehran, Iran.; ^8^Department of Endocrinology and Female Infertility, Reproductive Biomedicine Research Center, Royan Institute for Reproductive Biomedicine, ACECR, Tehran, Iran.; ^9^Department of Obstetrics and Gynecology, Arash Women's Hospital, Tehran University of Medical Sciences, Tehran, Iran.

**Keywords:** Sexual dysfunction, Prevalence, Meta-analysis, Women, Sex.

## Abstract

**Background:**

Cancer is one of the most common diseases and it has many physical and psychological consequences. Women with cancer are more likely to suffer from sexual dysfunction (SD) than healthy women.

**Objective:**

To estimate the overall prevalence of SD in women with cancer.

**Materials and Methods:**

The international databases Google Scholar, Embase, PubMed, Web of Science, and Scopus were searched for related articles without any time limitation. The keywords “Neoplasia”, “Tumor”, “Cancer”, “Malignancy”, “Female Sexual Function Index”, “FSFI”, and “female sexual dysfunction” along with their combinations were used in the search. Inconsistencies in the data were examined using the I^2^ test. The data were analyzed using the meta-analysis method and the random-effects model in the Stata software.

**Results:**

The analysis of 24 articles with a sample size of 5483 women showed that the prevalence of SD in women with cancer was 66% (95% CI: 59-74%). The highest and lowest prevalence were in Africa and Europe, respectively (75%; 95% CI: 66-83% vs. 43%; 95% CI: 26-60%, respectively). There was no relationship between the prevalence of SD and the mean age of the women, sample size, yr of publication, or quality of articles.

**Conclusion:**

SD is highly prevalent in women with cancer. African and American women with cancer have a higher average SD prevalence than Asian and European ones.

## 1. Introduction 

Cancer is one of the most widespread concerns of human beings, affecting both their emotional and physical wellbeing (1). It is projected that in 2028, over 1.7 million people will be living with cancer and over 600,000 of these people will lose their lives (2). Fortunately, nowadays many cancer patients survive due to timely diagnosis, accessibility of new treatments, and improvement in support systems (3). According to the National Cancer Institute, the rate of five-yr survival increased from 58% in 1975 to 83% in 2013 (4). It is expected that by 2024, there will be over 9.5 million survivors of cancer (5). Therefore, more attention should be paid to the problems of people with cancer, including quality of life considerations and complications resulting from late diagnosis. Among these concerns, sexual desirability has been an important point requiring sober and meticulous attention (1).

Sexual dysfunction (SD) has been categorized into four classifications encompassing problems related to arousal, interest, orgasm and pain (6). Female SD (FSD) in cancer patients is multidimensional, and includes factors related to psychology, physiology and sociology (7). The type of cancer and its treatment approaches (including chemotherapy, radiotherapy, surgery etc.) play an essential role in SD. Hence, SD in women with cancer is distinctively different from other women's impairments (8). One study revealed that females who underwent post-treatment chemotherapy had a higher risk of SD in comparison with those patients receiving no chemotherapy (9).

The insufficient attention given to sexual problems leads to negative impacts on couples' bilateral and mutual relationships (10). In addition to the time limitations of medical examinations, the reluctance of physicians and nurses to research this aspect of life, as well as the embarrassment of asking about sexual issues, create further obstacles to addressing SD (7). The effort to expand the focus on FSD is limited due to lack of sufficient screening tools and valid instruments (11).

The female sexual function index (FSFI) is one of few valid and reliable measurements to assess sexual-related problems among women with and without cancer (12). FSFI is a six-dimension tool by which female performance indices over the past four wk, such as desire, arousal, lubrication, orgasm, satisfaction and pain, are assessed through 19 questions. A score of 26 is considered the cut-off, and scores above this indicate satisfactory sexual performance (13). Data from studies about FSD among cancer survivors are highly heterogeneous.

The aim of this research was to examine the prevalence of SD in females with cancer through a systematic review and meta-analysis.

## 2. Materials and Methods

The present research utilized the meta-analysis of observational studies in epidemiology (MOOSE) checklist for reporting systematic reviews and meta-analyses (14). All scientific articles concerning SD in women with cancer were collected in January 2019. To do so, the following databases were systematically searched: PubMed, Web of Science, Google Scholar, Embase, and Scopus. The following keywords were used: “Neoplasia”, “Tumor”, “Cancer”, “Malignancy”, “Female Sexual Function Index”, “FSFI”, and “female sexual dysfunction”, using coordinating conjunctions “AND” and “OR”. The reference lists of articles were also searched to find additional articles not found through the initial searching process. Two researchers independently performed the search, extraction, and quality analysis of the studies. To be selected, the articles had to meet the following criteria: females with a cancer diagnosis were studied, FSD was a primary or secondary outcome, FSFI was used to evaluate the scope of SD, and published in the English language in a peer-reviewed journal. Unpublished full articles, posters, conference abstracts, letters to the editor, case reports and commentaries were excluded. Studies that did not report a total score, studies with inadequate data, studies that used only one dimension of the FSFI, studies with a sample size of less than 50 people, articles without full text, and studies published in non-English languages were excluded.

The name of the first author, yr of article publication, cancer type, design of the study, FSFI scores, country where the study was performed, and total number of patients were extracted from the articles. The methodological quality was evaluated by two independent reviewers in compliance with the Newcastle–Ottawa Scale. In case of disagreement, a third party's assessment prevailed. The articles were graded with stars from 0 (the poorest quality) to 9 (the highest quality).

### Statistical analysis

We calculated the variance of the prevalence of SD in each of the selected articles based on the binomial distribution formula. Heterogeneity was assessed using Cochran's Q test and I^2^ statistics and due to the high heterogeneity, a random-effects model was used to combine studies and estimate the pooled prevalence. According to the I^2^ index, heterogeneities were divided into three categories: low ( < 25%), medium (25 to 75%) and high ( > 75%) (13). To ensure the stability of the results, a sensitivity analysis was performed so that the pooled prevalence was assessed by omitting each study to identify a study whose omission had a significant effect on the pooled prevalence. To investigate the relationship between the prevalence of SD with the mean age of women, the yr of publication, the sample size of the selected studies, and quality score, meta-regression was used. Also, with subgroup analysis, the prevalence of SD was estimated by geographical area. A funnel plot based on Egger's regression test was used to evaluate the publication bias. Data analysis was performed using the Stata software (version 11).

## 3. Results

**Figure 1:**
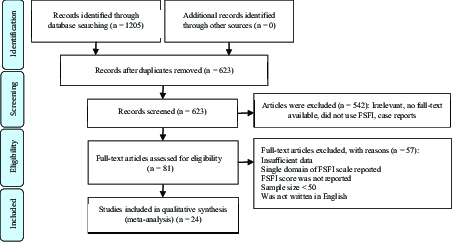
PRISMA-based selection and screening process.

A PRISMA flow diagram of the search strategy is illustrated in figure 1. The first attempt to search identified 1205 articles. 582 duplicate articles were removed. In the next stage, the individual papers were investigated based on title and abstract and 542 articles were excluded due to not conforming to the inclusion criteria. Then, 81 full-text papers were studied. 24 studies were included in the final analysis, of which 17 were cross-sectional, four were prospective studies, two were retrospective studies, and one was a case-control study. 11 studies (15-25) addressed breast cancer, one study colorectal cancer (26), four studies cervical cancer (27-29), three studies endometrial cancer (30-32), one study ovary and uterus cancer (33), and one study rectum cancer (34). Two studies (35, 36) dealt with patients categorized based on different cancer sites. The sample size ranged from 59 patients (26) to 1071 patients (27). Three studies were conducted in developing countries (15, 18, 22) and others were performed in developed countries (Table I and Figure 1).

**Figure 2:**
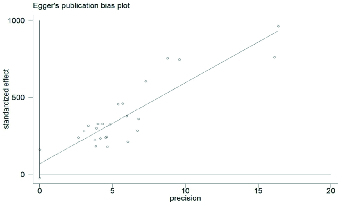
Publication bias.

To decide whether to include all of the articles examining SD or not, a publication bias chart was created. The results showed that publication bias was not significant (p = 0.20) (Figure 2).

**Figure 3:**
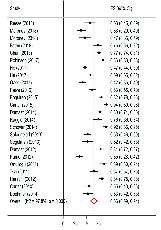
Prevalence of FSD in women with cancer based on random-effects model.

The results of the sensitivity analyses showed that the elimination of each article bore no effect upon the prevalence of the disorder. Pooled prevalence of FSD in patients was 66% (95% CI: 59-74%). Most of the studies were conducted in the United States of America. The findings of the subgroup analyses demonstrated that the prevalence of SD in the continents of Africa, America, Asia, and Europe was 75% (95% CI: 66-83%), 68% (95% CI: 61-75%), 62% (95% CI: 48-75%) and 43% (95% CI: 26-60%), respectively (Figure 3).

**Figure 4:**
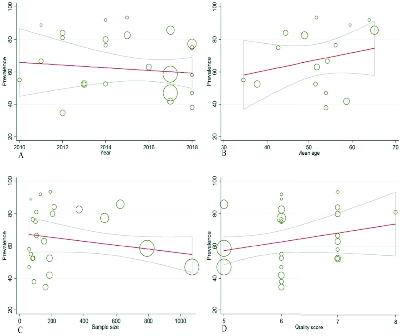
Meta-regression diagram of the prevalence of FSD based on: Yr (A), Mean age (B), Sample size (C), Quality score (D).

Regarding the type of cancer, the subgroup analysis showed that the highest and lowest prevalence of FSD was in women with genital and colorectal cancers, respectively. Meta-regression revealed that the prevalence of FSD bore no relationship with publication yr (p = 0.40), mean age of the women (p = 0.31), sample size (p = 0.97), or article methodological quality score (0.38) (Figure 4).

**Table 1:** The characteristics of the selected papers


**Author, yr (Ref)**	**Country**	**Design**	**Sample size**	**Mean age of women (yr)**	**Cancer type**	**FSD prevalence (%)**
**Reese ** ***et al.*** **, 2018 (26)**	USA	Prospective	59	- Colorectal	58
**Moroney ** ***et al.*** **, 2018 (35)**	USA	Cross-sectional	91	54.4 ± 12.3	Multiple	38
**Moroney ** ***et al.*** **, 2018 (35)**	USA	Cross-sectional	60	54.0 ± 12.2	Multiple	47
**Faten ** ***et al.*** **, 2018 (15)**	Tunisia	Cross-sectional	100	42.6 ± 6.9	Breast	75
**Chan ** ***et al.*** **, 2018 (33)**	USA	Cross-sectional	529	- Ovary-Uterus	77.3
**Robinson ** ***et al.*** **, 2017 (16)**	Canada	Prospective	625	- Breast	85.7
**He ** ***et al.*** **, 2017 (27)**	China	Retrospective	1071	- Cervical	47
**He ** ***et al.*** **, 2017 (27)**	China	Retrospective	792	- Cervical	58.6
**Gass ** ***et al.*** **, 2017 (17)**	USA	Cross-sectional	186	58.7	Breast	42
**Paiva ** ***et al.*** **, 2016 (18)**	Brazil	Cross-sectional	153	51.9 ± 9.2	Breast	63
**Boquiren ** ***et al.*** **, 2015 (19)**	Canada	Cross-sectional	127	49.0 ± 7.9	Breast	82.5
**Carter ** ***et al.*** **, 2015 (36)**	USA	Cross-sectional	190	51.8 ± 10.4	Multiple	93.5
**Damast ** ***et al.*** **, 2014 (30)**	USA	Cross-sectional	205	- Endometrial	80
**Raggio ** ***et al.*** **, 2014 (20)**	USA	Cross-sectional	83	56.2	Breast	76.5
**Schover ** ***et al.*** **, 2014 (21)**	USA	Cross-sectional	129	63.9	Breast	92
**Safarinejad ** ***et al.*** **, 2013 (22)**	Iran	Cross-sectional	186	37.7	Breast	52.5
**Segelman ** ***et al.*** **, 2013 (34)**	Sweden	Cross-sectional	82	- Rectum	52.4
**Damast ** ***et al.*** **, 2012 (31)**	USA	Cross-sectional	104	- Endometrial	81
**Pumo ** ***et al.*** **, 2012 (23)**	Italy	Cross-sectional	162	- Breast	34.7
**Onujiogu ** ***et al.*** **, 2011 (32)**	USA	Cross-sectional	72	59.5	Endometrial	89
**Tsai ** ***et al.*** **, 2011 (28)**	Taiwan	Cross-sectional	105	54.3	Cervical	66.6
**Harirchi ** ***et al.*** **, 2012 (24)**	Iran	Prospective	216	44.3	Breast	84
** Author, yr (Ref)**	**Country**	**Design**	**Sample size**	**Mean age of women (yr)**	**Cancer type**	**FSD prevalence (%)**
**Carter ** ***et al.*** **, 2010 (29)**	USA	Prospective	71	34.5	Cervical	55
**Boehmer ** ***et al.*** **, 2014 (25)**	USA	Case-control	85	51.6	Breast	52.5
FSD: Female sexual dysfunction						

## 4. Discussion

This study presented a meta-analysis of the literature on cancer and FSD. The purpose was to study and address the problems of SD in women. We only included observational studies that evaluated sexual function with a validated test. Sensitivity analyses showed that the results were strong, and there was no significant diffusion bias. The results of meta-regression showed that neither FSD prevalence nor sexual function scores in women with cancer were affected by the mean age of the women, yr of publication, sample size, or quality of score.

The findings of this study showed that two-thirds of these patients (66%) suffered from SD, which was similar to the findings previously reported ( > 60%) by Maiorino et al. (37).

Other studies have shown that upon starting treatment, 40% of women with cancer suffer from SD and 50% of breast cancer patients experience SD long term (38, 39). Another study revealed that cancer and its treatment can have a direct and conclusive impact on desire, arousal, orgasm, and dyspareunia, and that the severity of SD and how long it lasts depend on radiotherapy and its duration (40).

Lawman et al. reported that 43% of American female patients in their study experienced FSD, while approximately 63% developed FSD in a study conducted in mainland China (39, 41). A study conducted in Iran on 100 women with cancer showed that 60% of patients had moderate SD (42). Anderson adds that surgery on patients with cancer, such as hysterectomy, mastectomy and ovarian resection, can significantly impair sexual function, which can threaten the quality of life in women with cancer (43).

The findings of one study showed that the prevalence was highest in women with gynecological cancer, so that in a meta-analysis, female dysfunction was reported as the highest rank (78%) (37). Survivors of gynecological cancers have been found to experience SD at higher rates than survivors of other cancers (44). In addition, gynecological cancer treatments usually involve surgery, which can be combined with radiation therapy, chemotherapy, and hormone therapy that often have direct effects on sexual organs (45).

Gynecological issues and sexual function for women with cancer are considered great challenges, originating from patient social status and disease impacts, as well as side effect complications (46). Cancer type and treatment modalities have an impact on FSD. These distinctions partially affect FSFI scores (47). One study indicated that the most prevalent dysfunctions among women with cancer are loss of desire for sex (39%), vaginal dryness (24%), dyspareunia (9%), difficulty feeling excitement and pleasure (21%), and issues in orgasm (15%) (48). Vaginal spasm and dryness can be due to the effects of chemotherapy drugs and a lack of sexual arousal in these women. Also, lack of stimulation prevents the secretion of viscous vaginal fluid and so leads to painful intercourse (49). In these women, constant mental preoccupation with the defect may cause low stimulation or even lack of stimulation (50, 51). Frederickson and Robert point out that when people are distracted by their appearance, they cannot focus on their sexual pleasure, and this negatively affects their sexual function (49). Estrogen levels drop naturally during chemotherapy. It is well known that a lack of estrogen results in atrophy of the urogenital system and dyspareunia, lowering sexual desire and arousal (48, 52). Given these findings, it is important to pay more attention to changes in the sexual function of women with cancer. Thus, these results can help to increase the availability of assistance to women.

The subgroup analyses showed that most articles on FSD have been published in the United States. Most valid tools for measuring FSD are designed by Americans, are in English and are tailored to the American culture (53). Sexuality is a concept based on cultural norms and influences. In other words, cultural factors determine the importance and meaning of sexual behaviors. Therefore, sexual issues should be discussed within the framework of the prevailing culture of that society (54). For this reason, the World Health Organization recommends explaining the concept of sexual health in a cultural context (55). Sex has always been a taboo subject especially in Asian society. This has led researchers in Eastern countries to focus less on sexuality, especially in women. These countries often use translated tools that are not based on their own culture and historical background, which creates problems in collecting data and interpreting results. In his review, Lewis showed that because there are fundamental methodological problems in data collection, there are no articles comparing sexual performance in Asia with other parts of the world. In addition, there are few studies on the incidence of SD in Asian populations (53).

Our findings showed that FSD is more prevalent in American and African women with cancer than in Asia and Europe. Ethnic, social or cultural differences seem to be the reason for this finding. Therefore, more studies are needed to confirm the reliability of these regional differences.

The present research focused on the FSFI because it is one of the only valid instruments for cancer survivors, and it was created by the National Comprehensive Cancer Network (56) and the Patient - Reported Outcomes Measurement Information System (PROMIS) committee (11). A widespread epidemiology study on 7243 healthy females with ages ranging from 40 to 59 was conducted to evaluate FSFI scores which resulted in a FSFI score of 25.2 (57). However, the average score of FSFI in middle-aged women can be lower than the cut-off on account of sexual performance dysfunction, menopause, and hormone and metabolism-related changes (58).

At present, there are some tools for evaluation of SD in cancer patients such as the Sexual Function-Vaginal Changes Questionnaire (59) and the sexual function after treatment for gynecologic cancer tool (60). Some tools are sex-specific (i.e., Derogatis Sexual Functioning Inventory (61) and Changes in Sexual Functioning Questionnaire (62)). Furthermore, numerous site-specific measurements have been developed to assess cancer patients' quality of life, with function or symptom subscales for sexual function assessment (e.g., the European Organization for Research and Treatment of Cancer quality-of-life questionnaires (63)). Most of these tools encompass a few items in a general scale to assess SD. Accordingly, they deal with the general quality of life of cancer survivors (63).

It is noted that the sample sizes of most surveys are small. We excluded research with fewer than 50 subjects as well as those studies with questionnaires other than the FSFI. A limitation of our study is that studies which were written in languages other than English were omitted. We also did not perform subgroup analyses for the clinical stage, treatment regimen, or other possible factors that may have affected the results.

## 5. Conclusion

Overall, our results confirm that the prevalence of FSD in women with cancer is high. Women with cancer in Africa and the United States have a higher prevalence of FSD than those in Asia and Europe. Since the sexual needs of women with cancer are different from those of healthy women, the development of a dedicated, specialized tool to evaluate the FSD of women with cancer is necessary.

##  Conflict of Interest

The authors declare that there is no conflict of interest.
